# A Plea for More Interest in Prosthetic Dentistry

**Published:** 1906-08

**Authors:** H. R. Jewett

**Affiliations:** Atlanta, Ga.


### A PLEA FOR MORE INTEREST IN PROSTHETIC DENTISTRY.

By H. R. Jewett, D. D. S., Atlanta, Ga.

(Florida State Dental Society, 1906.)

The history of dentistry as a science and profession, like that of the world, of nations, and of individuals, is marked by epochs. While in ancient history we find very little of the practice of dentistry as a profession, yet several writers, both sacred and profane, have made sufficient reference to the teeth, and work on the same, to lead us to believe that the practice of dentistry *might* be as old as history itself.

The first reference to a material used in dental work in the Holy-Book is the precious metal, gold, just after the creation of Eve.

Just what the object of the Creator was in directing Adam, immediately after the creation of woman, where he would find gold in abundance I am unable to say. Nor would I even conjecture that the precious metal to which Adam was directed, was to be used for dental purposes; since it was evident, that after the spring and summer had past, that they were then spending in the beautiful garden, and after they had been thrust out on the cold world, the foliage of the trees and the fig leaves would be shriveled by the chilling frost, making shopping for mother Eve necessary in other quarters; which might prove exceedingly embarrassing to the good lady, unless her sole reliance, Adam, had supplied her abundantly with filthy lucre-gold. It might however, be safe to say, that after this awful judgment had been passed on them a department store would more likely be needed, than a dental office. We do have the mention of dental work, the use of gold in the mouth, etc., by Plinney, and by other writers many years before the Christian era. In a way, too, that would indicate prosperity, as well as operative work. These historical statements are merely suggestive of crude means and methods of the practice of dentistry, hundreds of years possibly before Christ, and with no evidence of any marked improvement at any special period. Not until less than a century ago, can we find that the door to the forward movement of the advancement of dental surgery was unbarred. In fact, the doors to progress were never open, until that noted period of the establishing of Dental Societies-Dental Journals-and Dental Colleges. Since then, there has been made some wonderful strides, both in methods and results in the practice of dentistry. Recall with me some of these. Think of the wonderful successes of the use of soft foil in the hands of such skilled operators as Hudson, Harris, Townsend, Clark, Morgan, Crawford and others, and also the use of tin-foil, and combination of tin and gold. But the work of *gold* foil was limited, until the discovery of the cohesiveness of gold by Arther, which discovery, by the skillful manipulation of such men as Barnum, Varney, Webb, McKellops and others, gave contour and restorative work such an impetus, as to bring it down to the present, face to face with the esthetic porcelain work and combination filling of all kinds. During this time, we have also been introduced to the many plastic-fillings, each serving their purpose, and helping to give a steady advance in the progress of operative dentistry.

As to appliances, leaving the old methods of preparing cavities and manipulating gold-foil with hand instruments, we strike into a successon of improved implements and appliances and electric machinery too numerous and familiar to you all to consume your time in mentioning. Yet each has played its part in keeping operative dentistry on the steady march of improvement.

This brings us to the heart of the subject of this paper. Has *prosthetic* advanced, hand in hand, with operative? Is the interest manifested alike in the one as in the other? On the contrary has not the progress in prosthetic dentistry been more by spasmodic moves, each stage of which, being an epoch, marked by its want of onward movement, and of indifference to the quality of its work? The different stages or epochs of prosthetic dentistry might be named as follows; Gold and Silver plates—Continuous-gum—Rubber-plates—Celluloid work—Bridges-back to rubber work—Laboratories—Cheap-laboratories. Does this not look more like progress backward? It does sometimes seem a little hard to tell whether, in this department, we are coming or going.

If this be true, where lies the fault? Surely not for the want of improved implements and appliances, or of a scarcity of literature on this subject, as the mere mention of the names of such men as Bonwell, Kingley, Evans, Kirk, Essig and others suggestive of many of the implements and appliances, and of the abundance of literature on this subect will prove.

My ambition and my desire to see this branch of dentistry—Prosthetic—keep well abreast with every other department of the profession, may sometimes lead me to draw wrong conclusions, but it does strike me that the interest in this line is not as manifest, nor the general results in practice as satisfactory, as in some other deparments. If I am correct, then where lies the fault? Here I lay myself open to attack by assigning as one of the causes, the large number of cheap laboratories, and a generous disposition on the part of so many dentists to encourage and support them by a very liberal, and unnecessary patronage. I know there are cases where dental laboratories serve as a great help to some dentists. They have their place, and for that reason I use the expression, unnecessary patronage, which means that many patrons of these laboratories rely too much on the laboratory men to do the work that ought *always* to be done at the chair, the patient being present. For instance, a dentist will take an impression and bite, forward the same to a laboratory with the instructions to make and send plate by return mail with special delivery stamp. Tell me that the patient has in this case had the benefit of a careful, painstaking, skillful, artistic dentist? What does the laboratory man know about the temperament, age, general appearance, etc., of the patient? All of which has so much to do with the selection and arrangement of the teeth. Who of you have not received circulars from cheap-John laboratories far away as the extreme northern, eastern, and western parts of the country, soliciting patronage with schedule of prices for crowns and bridges, for less than what the gold would cost *you* for the same work *properly* made ? They evidently get a very liberal patronage at these prices, or business could not keep up. Would you be suprised at any time to find this P. S. to one of these soliciting circulars? "We also sell gold dollars for ninety-five cents, and pay postage both ways."

My next assertion may be challenged by some. If so, it's your privilege to stir up the hornet's nest. A large number of dentists send to laboratories to have work done, especially crown and bridge work, who cannot do the work themselves, and a dentist who cannot make a crown or bridge, certainly could not properly prepare the teeth to receive it. The assertion is a general and broad one consequently, not without its exception, so I leave it for your criticism or discussion.

Did you ever consider for a moment how much broader is the field in prosthetic dentistry for a display of real artistic skill, and for giving absolute service to the patient, than is in operative dentistry? This skillful and artistic work must be done with the patient seated in the chair, before you, and cannot be done in a laboratory hundreds of miles away. The nice smooth finish, and polish of gold fillings suggest, and sometimes *convince* patients that they have a very satisfatory and durable piece of work. This finish and polish is done by the same operator who *knows* that his skill, and hope of real service done the patient is more in the preparation of the cavity and the insertion of the filling, than in the polish it afterwards receives.

The smooth finish and polish on the plates, crowns and bridges you receive from the laboratories, that attract your attention quite as much as "the shine" of the gold filling does that of the patient, is in most cases, done by the little negro office boy. It may appear harsh and unkind in me to accuse a brother dentists of allowing the finish and polish of this class of work to overshadow the more important detail, but *I know*, in many instances, as I have learned from conversations with dentists patronizing dental laboratories, that my assertion is true. And I think I am fully justified in believing, that the many little details, worth so much to the patient, where the work is done at the chair, could be easily lost in transit from a dental office to a laboratory, hundreds of miles away. And I am equally justified in believing that the dentist who gives over the real artistic part of his prosthetic work to another, soon loses interest in it himself; which amounts to a retrograde, instead of advancement, and both he and his patients will suffer thereby.

Some other points, closely connected with this subject, I will only call attention to. The tendency of dentistry to divide into branches on specialties—Operative, Prosthetic, Orthodontia, etc. —would such a division give better results and better prices, for the dentist, and give better service to our patients? Would the interest of the profession and advancement in each department be greater than at present? Would a prosthetic specialist rely on some dental laboratory to do his work? Do dental laboratories encourage or discourage special interest in prosthetic work?

No part of this paper is intended as an attack on dental laboratories. I do say, however, that I look upon cheap advertising dental laboratories, just as I do cheap "dental parlors." I know of some dental laboratories that are run on the highest plane of dental ethics, and are of great service to quite a number of dentists who use them in the right way; but I do criticise the dentist who takes the impression and bite, leaving the remainder of the work to some laboratory man, and question if his patients do not suffer for the want of *professional* service.

